# Power and resistance in schools: Implementing institutional change to promote health equity for sexual and gender minority youth

**DOI:** 10.3389/frhs.2022.920790

**Published:** 2022-11-29

**Authors:** Daniel Shattuck, Bonnie O. Richard, Elise Trott Jaramillo, Evelyn Byrd, Cathleen E. Willging

**Affiliations:** ^1^Pacific Institute for Research and Evaluation, Southwest Center, Albuquerque, NM, United States; ^2^Pacific Institute for Research and Evaluation, Louisville Center, Louisville, KY, United States

**Keywords:** implementation science, power, health disparities, sexual and gender minority, heteronormativity, LGBTQ adolescents, school health

## Abstract

**Introduction:**

Schools in the United States are hierarchical institutions that actively (re)produce the power relations of the wider social world, including those associated with heteronormativity. Structural stigma, informed by heteronormativity and perpetuated through schools, contributes to the production of academic and health disparities among youth who are lesbian, gay, bisexual, transgender, queer, or of other gender and sexual identities (LGBTQ+). We draw upon 5 years of qualitative data from a cluster randomized controlled trial conducted in New Mexico that used implementation science frameworks to promote the uptake and sustainment of evidence-informed practices (EIPs) to examine how power operates to hinder or promote the ability of school staff to change school environments, disrupt structural stigma, and increase safety and support for LGBTQ+ youth.

**Methods:**

Data sources included annual individual and small group qualitative interviews with school professionals (e.g., administrators, school nurses, teachers, and other staff), several of whom took part in Implementation Resource Teams (IRTs) charged with applying the EIPs. Other data sources included bi-weekly periodic reflections with implementation coaches and technical assistance experts. Data were recorded, transcribed, and analyzed using deductive and inductive coding techniques.

**Results:**

The IRTs experienced variable success in implementing EIPs. Their efforts were influenced by: (1) constraining school characteristics, including staff turnover and resource scarcity; (2) community-based opposition to change and concerns about community backlash; (3) the presence or absence of supportive school leadership; and (4) variations in school, district, and state policies affecting LGBTQ+ students and attitudes about their importance. Findings illustrate how diverse power structures operated in and across outer and inner contexts to bound, shift, amplify, and otherwise shape how new practices were received and implemented.

**Conclusion:**

Findings indicate that the efforts of IRTs were often a form of resistant power that operated within and against school hierarchies to leverage epistemic, discursive, and material power toward implementation. To improve health equity, implementation scientists must attend to the multiple real and perceived power structures that shape implementation environments and influence organizational readiness and individual motivation. Implementers must also work to leverage resistant power to counter the institutional structures and social norms that perpetuate inequities, like heteronormativity and structural stigma.

## Introduction

Schools are institutions that both mirror the power relations of the wider world and actively (re)produce them, including norms and behaviors informed by heteronormativity, meaning the assumption that heterosexuality and cisgender identity are the most normal and natural state of human sexuality and gender (1–5). Heteronormativity is communicated in schools overtly through the presence of homophobic and transphobic language, bullying behavior, gendered dress codes, and rules prohibiting “public displays of affection” (6–11). Heteronormativity is also communicated covertly through school spaces (e.g., gendered restrooms), policies and practices (e.g., dividing sports into boys' and girls' teams), and widely shared values (e.g., beliefs that sexual orientations and gender identities are irrelevant because all students should be treated the same) (12–14). School environments thus perpetuate structural stigma, referring to the mechanism through which institutional policy and practice and larger societal norms erase, discriminate, and victimize LGBTQ+ populations (15–17). By decreasing or foreclosing social safety, which is manifest through social connection, inclusion, and protection (18), and allowing minority stress experiences to occur (19), structural stigma contributes to negative mental, physical, and academic outcomes for LGBTQ+ populations (16, 20–23). These outcomes include dropping out of school (24), self-harm (25, 26), substance use (27, 28), sexually transmitted diseases (29, 30), and poor mental health (31, 32).

Conversely, schools can bolster LGBTQ+ health by improving social safety and decreasing minority stress through practices that resist heteronormativity and disrupt structural stigma. The Centers for Disease Control and Prevention (CDC) identify six practices that improve school culture and climate for LGBTQ+ youth (33). These practices include (1) identification of safe spaces on campus, such as Genders and Sexualities Alliances (GSAs), where LGBTQ+ youth can receive support from administrators, teachers, other school staff, and peers; (2) prohibition of harassment and bullying based on sexual orientation or gender expression; (3) provision of health education curricula inclusive of LGBTQ+ youth; (4) professional development of staff on safe and supportive school environments; (5) facilitation of access to medical providers experienced in serving LGBTQ+ youth; and (6) facilitation of access to behavioral health providers experienced in serving this population.

Mounting evidence demonstrates that these school-based supportive practices can positively affect the health and wellbeing of LGBTQ+ young people. The presence of safe spaces, GSAs, non-discrimination policies, inclusive curricula, and affirming school staff is associated with reduced homophobic victimization (34, 35), increased school belonging (36), and perceptions of greater safety at school (37–40). These improvements in social safety and reductions in minority stress experiences lead to decreases in risk behaviors (41, 42), improved academic outcomes (43, 44), and better mental health (45, 46). Finally, schools are key sites for prevention, screening, treatment, and referral to healthcare services for LGBTQ+ and other underserved youth who would otherwise face barriers to accessing appropriate, competent, and affirming care (47–49). There is thus a strong public health need to enact institutional change in schools to disrupt the processes of structural stigma and cultivate environments that are affirming, supportive, inclusive, and explicitly protective of LGBTQ+ populations (12, 47, 49, 50).

At the same time, schools are hierarchical and bureaucratic systems in which control and use of power pose challenges to implementing new practices. For example, the priorities set, decisions made, and resources directed by the upper levels of school administration shape school systems and limit the types of actions, activities, and behaviors allowed within their purviews. Recent work in implementation science that draws upon the writings of philosopher Michel Foucault showcases a typology of power that can be generated and leveraged in implementation practice. The three types are discursive power (e.g., the power to frame problems), epistemic power (e.g., the selection and use of knowledge), and material power (e.g., control of resources like funding and staffing) (51). These three types and their interplay enable and constrain possibilities for implementation efforts; importantly, not all stakeholders are equally able to wield each type. Just as the use of power can fulfill a dominant function (maintaining status quo) or a resistant function (enabling change), the same stakeholders in implementation contexts may wield power for both dominance and resistance (2, 3). Initiating institutional change to improve health equity for LGBTQ+ youth thus requires operating within the complex webs of power comprising school environments and considering the constraints and possibilities afforded to staff and students through the control over discursive, epistemic, and material power (52, 53).

For the present analysis, we examine the role of power in the work of school professionals to implement the six CDC-identified LGBTQ+ supportive evidence-informed practices (EIPs), outlined above (54). Our central question is: How does power operate to hinder or promote the ability of school staff to change school environments to increase safety and support for LGBTQ+ youth? Ultimately, we interpret the work of school staff in carrying out the EIPs as an exercise of resistant power against structural stigma and the dominant powers exercised through school and community social hierarchies.

### Study background

We conducted a 5-year cluster randomized controlled trial in 42 secondary schools across the rural state of New Mexico (54). Entitled “Implementing Strategies to Reduce LGBTQ+ Adolescent Suicide” (RLAS), the study examined the uptake and sustainment of the six CDC-identified EIPs for improving school support, safety, and mental health for LGBTQ+ youth. The Exploration, Preparation, Implementation, and Sustainment (EPIS) framework guided EIP implementation. EPIS is a four-phased implementation framework that emphasizes the careful examination of outer and inner contexts and bridging factors during the Exploration phase to inform future activities in the Preparation and Implementation phases (55, 56). Outer contexts represent the higher levels of influence on schools (e.g., school districts, communities, and state educational systems), such as legislation and district policy, community-level advocacy, stigma, and funding (54, 55, 57). Inner contexts encompass the internal environments of schools, including physical and social organization and the attitudes and practices of staff and students (55). Important inner-context factors are school readiness for change, perceived need to change practices, school and staff values, and attitudes toward EIPs. Bridging factors that connect the outer and inner context include community-academic partnerships, coaching support, and formal contracts or memoranda of understanding (58).

We paired EPIS with the Dynamic Adaptation Process (DAP), an iterative data-informed methodology for tailoring each step of EIP implementation to the specific school-community contexts (59). Central to the DAP was the formation and training of Implementation Resource Teams (IRTs) in each school, comprised of administrators, teachers, staff, and occasionally students. These IRTs were charged with local assessment, planning, and implementation of the six EIPs, and supported by an implementation coach.

We invited 145 New Mexico public high schools to take part in RLAS. As described elsewhere (54), school eligibility required student participation in the New Mexico Youth Risk and Resilience Survey (NM YRRS), the state's extension of the CDC's Youth Risk Behavior Surveillance System, for outcome monitoring purposes. Additional inclusion criteria were the presence of a school professional (e.g., school nurse, librarian, teacher) and a high-ranking school administrator willing to support EIP implementation. However, the relative autonomy of schools to participate varied across the state. For example, some districts required the approval of their internal research review boards, whereas others required approval from a superintendent, principal, or both. Administrators of schools declining participation cited general impediments (e.g., limited time and staffing), concerns about fomenting negative reactions in conservative communities, assumptions that there were no LGBTQ+ students on campus, and the idea that LGBTQ+ students did not warrant specialized interventions (60). We later discovered that such beliefs were also present in the schools that enrolled in RLAS (57). In the end, we recruited 42 schools.

After randomization into implementation and delayed implementation conditions, we assessed the baseline capacity of participating schools to implement the EIPs, including school needs, facilitators, and barriers (54). Schools in the implementation condition received tailored guidance and support from trained coaches for 3 years, followed by a 1-year sustainment period without coaching support. Those in the delayed condition received guidance and support from the coach in the final year of RLAS as the original implementation schools entered their sustainment phase. All schools received an annual payment of $500 to offset the costs involved in participation.

## Materials and methods

### Study context

We conducted RLAS in the largely rural and culturally diverse state of New Mexico, USA. The state ranks 46th in median household income and has the third-largest percentage of residents below the poverty level (18.4%) in the nation (61). Hispanic/Latinx and Native American people comprise 60% of residents (62). In New Mexico, about 5.9% of adults (63) and 14.5% of high-school students identify as sexual minorities (64); 0.67% of adults (65) and 3.2% of high-school students identify as gender minorities (64). The 2019 NM YRRS found that nearly half of the youth identifying as LGBTQ+ had experienced non-suicidal self-injury, symptoms of depression, or suicidal ideation in the past year (64). Further, almost a quarter of sexual minority youth and a third of transgender youth had attempted suicide in the past year (64). When we initiated RLAS in 2016, only 17% of secondary schools in New Mexico had implemented all six of the focal EIPs (66).

### Data collection

Between 2016 and 2021, we collected qualitative data annually with key staff from participating high schools. Each year, we invited the IRT leads and administrators from each school to participate in 60-min semi-structured interviews and IRT members to take part in a 90-min small group interview. Across the 5 years of data collection, a team consisting of five anthropologists and two master's level research assistants conducted the interviews, which were audio recorded and professionally transcribed. Questions in the semi-structured interview guides focused on implementation efforts and power structures. In addition, they examined knowledge of and comfort with LGBTQ+ youth, efforts to implement EIPs, and additional relevant outer-context factors per the EPIS. Table 1 describes the samples for the annual interviews. Bi-weekly debriefing meetings with study coaches, research staff, technical assistance experts, and principal investigators facilitated ongoing discussions of unfolding events and themes. The Pacific Institute for Research and Evaluation Institutional Review Board approved the research protocols and informed consent procedures.

**Table 1:** RLAS participant sample size by interview type.

	**Y1 (2016–2017)**		**Y2 (2017–2018)**		**Y3 (2018–2019)**		**Y4 (2019–2020)**		**Y5 (2020–2021)**	
	**Imp**.	**Control**	**Imp**.	**Control**	**Imp**.	**Control**	**Imp**.	**Control**	**Imp**.	**Control**
IRT lead interviews	27	25	20	23	21	23	21	18	20	19
Administrator interviews	22	19	17	19	19	20	13	19	15	18
IRT member interviews	20	–	–	–	–	–	–	–	–	–
IRT member small group interviews	–	–	16	–	17	–	18	–	15	16

Imp., Implementation.

### Participants

**Figure 1:**
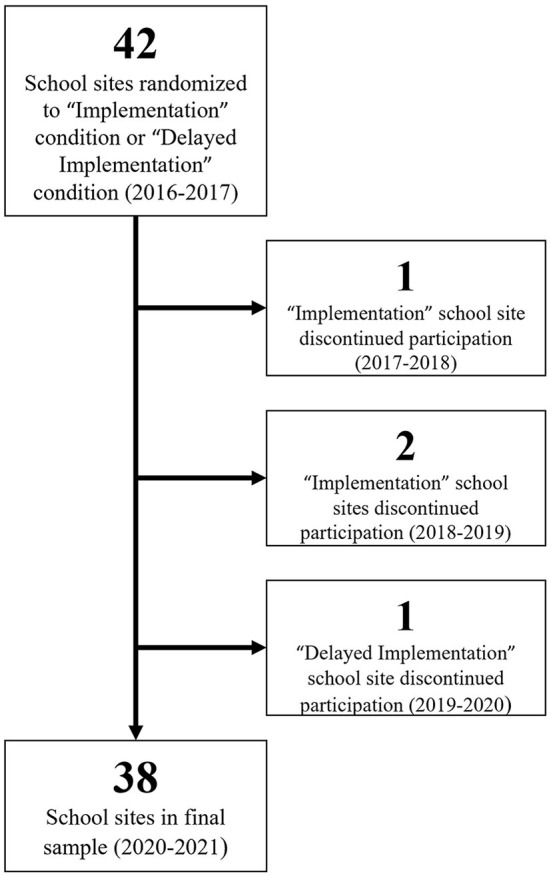
RLAS school sample over time.

Participating schools were located in rural, suburban, and urban communities across New Mexico. Interview participants included school administrators (e.g., principals and assistant principals) and the leaders and members of IRTs, including school nurses, social workers, counselors, teachers, librarians, and other staff. The final sample for this analysis included at least one IRT leader (*N* = 91), one administrator (*N* = 77) from each school, and a total of 132 IRT members. Over the study's course, four schools discontinued participation due to instability in staffing or turnover in leadership (Figure 1).

Approximately 68.3% of participants in our sample identified as female, 28.8% as male, 3.1% as gender diverse (e.g., trans man, trans woman, genderqueer/non-conforming, other), and <1% preferred not to answer. Participants reported their race separately from Hispanic/Latinx ethnicity. They described their races as white (69.6%), American Indian/Alaska Native (9.7%), Black (4.9%), Asian (1.0%), “other race” (14.6%), and “prefer not to answer” (4.2%). Many of the 44% of participants identifying their ethnicity as Hispanic/Latinx reported their race as “other race.” Table 2 includes participant demographics by study year.

**Table 2:** RLAS participant demographics.

	**Y1 %**	**Y2 %**	**Y3 %**	**Y4 %**	**Y5 %**	Overall %^*^
**Number of participants**	***N* = 114**	***N* = 141**	***N* = 151**	***N* = 117**	***N* = 153**	***N* = 309**
**Race (select all that apply)**						
American Indian, Alaska Native, Indigenous Latin American	9.6	11.3	7.9	6.0	7.8	9.7
African American, African Descendent, or Black	6.1	6.4	4.6	4.3	3.3	4.9
Middle Eastern American or Middle Eastern	0.9	0.7	0.0	0.0	0.7	0.6
Asian American or Asian	0.0	0.7	0.7	0.0	1.3	1.0
European American, White, Anglo, or Caucasian	71.9	59.6	68.9	69.2	64.7	69.6
Different race	17.5	16.3	19.9	5.1	5.9	14.6
Prefer not to answer	0.0	0.0	0.7	5.1	7.2	4.2
Missing	0.0	12.8	2.0	12.8	10.5	0.0
**Ethnicity**						
Hispanic	43.9	36.9	41.7	35.9	34.0	44.0
Not Hispanic	56.1	51.1	55.0	52.1	54.2	55.0
Prefer not to answer	0.0	0.0	0.7	0.0	0.7	0.3
Missing	0.0	12.1	1.3	11.1	10.5	0.6
**Current gender identity (select all that apply)**						
Female	65.8	61.7	64.9	59.8	62.7	68.3
Male	32.5	23.4	32.5	28.2	23.5	28.8
Transgender man/transman	0.0	0.7	0.7	0.0	0.0	0.6
Transgender woman/transwoman	0.9	0.7	0.0	0.0	0.7	0.6
Genderqueer/gender non-conforming	0.9	0.7	2.0	1.7	2.0	1.3
Different identity	0.0	0.0	0.7	0.0	0.7	0.6
Prefer not to answer	0.0	0.7	0.0	0.0	0.7	0.6
Missing	0.0	12.1	1.3	11.1	10.5	0.0
**Sexual orientation (select all that apply)**						
Bisexual	4.4	3.5	6.6	5.1	4.6	4.9
Heterosexual	87.7	73.0	80.1	70.1	72.5	80.9
Lesbian or gay	6.1	7.1	8.6	7.7	9.2	9.7
Queer	2.6	2.8	2.6	1.7	2.6	2.6
Questioning	0.0	0.0	0.0	0.0	0.0	0.3
Different orientation	1.8	0.7	2.0	1.7	1.3	1.9
Prefer not to answer	0.0	56.0	53.6	32.5	0.0	2.3
Missing	0.0	12.1	1.3	11.1	10.5	0.0

* Calculated based on all unique individuals involved with the study, unweighted by years of involvement.

### Analysis process

Four researchers, including two anthropologists and two master's level research assistants, conducted qualitative analysis using NVivo [Release 1.3 (535)], a qualitative data analysis application. The researchers iteratively coded professional transcriptions of interviews, compared between schools and participant types, and undertook targeted searches for specific concepts as needed. They applied deductive and inductive coding to identify themes and emergent patterns in the data, including inner- and outer-context variables and implementation status for each EIP. Example codes included “Safe Zones,” “LGBTQ 101,” “bathrooms, locker rooms,” and “access to behavioral health providers.” We maintained intercoder agreement during the routine review, discussion, and interpretation of coding output at biweekly team meetings.

To facilitate cross-time-point and cross-site analysis, we synthesized data from coded transcripts, implementation coach activity logs tracking engagement with school sites, and notes from debriefing meetings into comprehensive school reports. These extensive case summaries (67) chronologically described changes in the school-community environments between the baseline assessment period and the study's final year. Changes centered on the six focal EIPs, inner-context factors (e.g., school culture and climate, staff characteristics), and outer-context factors (e.g., family and community characteristics, state-level policy) according to EPIS phases.

We then analyzed school reports and engaged in yet another round of coding where we employed sensitizing concepts related to power, i.e., “dominant,” “resistant,” and “material” (68). Finally, we considered how the data relate to concepts of heteronormativity, how heteronormative discourses inform and are informed by school power structures, and how power is exercised to enable and constrain action within schools' hierarchical governance structures.

## Results

We organize our findings into four sections. First, we focus on school characteristics affecting the ability of IRTs to enact change in schools. This section illuminates the important role material power plays in implementation processes. Second, we discuss the dynamics described by participants as influencing their pursuit of changes, including the possible risks involved, expected, or encountered opposition, and the successes they experienced. These dynamics reflect how heteronormativity and structural stigma shaped the discursive power of decision-makers in schools. Third, we analyze how formal and informal leadership affected IRT efforts to implement EIPs, illustrating a web of power relations spanning outer and inner contexts and the usefulness of mobilizing formal hierarchies in schools to aid implementation. This section clarifies the influence of control over material power, the role of discursive and epistemic power in leveraging material power, and how leadership can serve as a mechanism for defending implementation efforts against power exerted by communities, parents, and staff to perpetuate structural stigma intentionally or unintentionally. Finally, we examine how formal authority imbued in policy can impact supportive intervention for LGBTQ+ youth. These findings demonstrate how participants thought about their own power to promote institutional change and the role of policy in perpetuating or disrupting structural stigma.

### Constraints of school characteristics

Two common and interrelated characteristics of New Mexico schools posed challenges by limiting the availability of material power for implementation: staff turnover and scarcity of resources. Participants explained that resource scarcity in terms of time constraints, low pay, high job stress, and living and working in underserved areas contributed to high turnover.

Turnover was a common problem for all our participating schools, regardless of geographical location. A small rural school exemplified these difficulties: even before the implementation period officially began, recently hired administrators and multiple staff who had agreed to serve on the IRT moved on from their job posts. This instability in staffing led the school to withdraw from the study.

School professionals elsewhere described being overextended due to staff shortages, clarifying that they could not fulfill their day-to-day job functions and fully support students. A nurse in an urban school explained, “I am alone a lot of times…. It's chaotic. I have these students that I wish I could spend more time with them and help, but I can't… Who can I call? I need a backup here.” Participants also highlighted the tendency for a limited number of personnel to take on extra work responsibilities. The self-selected composition of IRTs exemplified the issue, as most members served on other school committees (e.g., academic, attendance, student wellness, social and emotional learning) and as sponsors of student activities (e.g., clubs, athletics). Despite often being able to build on experience and cache accrued through such involvement to enable EIP implementation, these individuals struggled against feeling overwhelmed due to competing demands for their time and energy.

Participants ubiquitously commented on the scarcity of material resources (e.g., funding) that contributed to the staffing shortages and subsequent hardships in changing health curricula. For example, after IRT members donated their time to identify, vet, and obtain approval for a new LGBTQ+ inclusive health curriculum from their rural school's administration, the school struggled to maintain a health education teacher on staff long enough to implement it.

The physical environments of school buildings constrained efforts at several sites to implement best practices for creating safe spaces. For example, having gender-neutral restrooms was a noted source of tension, as there were few or no single-user restrooms—often the most feasible to modify from gendered to gender-neutral—available for students in most schools. In describing an IRT's progress in establishing gender-neutral restrooms, one school administrator disclosed, “We struggle with gender-neutral bathrooms, and it's not because we don't care. It's because of the age of our buildings. This building we're sitting in is as old as I am, and I'm 57. So, imagine that.” The administrator added, “We're a poor district,” and lamented the difficulties of adapting existing infrastructure and how resource scarcity foreclosed possibilities for change. The universal condition of scarcity among participating schools impeded material power to support EIP implementation by IRTs.

### Concerns about community perceptions and backlash

The changes prioritized by IRTs were influenced by speculations of whether school and community stakeholders would view them favorably. Participants in rural and suburban areas often described their communities as socially conservative, noting the omnipresence of “traditional” gender roles, religiosity, and low knowledge and awareness of LGBTQ+ populations. They described outright discrimination, prejudice, or ignorance to which they were privy or had experienced firsthand and cited concerns about parental disapproval of EIPs and potential social or professional repercussions. These concerns illustrated the power exercised by communities over schools; staff recognized how mechanisms of structural stigma (e.g., discrimination and violence) facilitated the exertion of this power. Both assumptions about community reactions and the lingering impacts of past experiences compounded with the difficulty of initiating projects in schools and created hesitation among staff. In some schools, concerns about community pushback led IRTs to focus on EIPs that members perceived as less controversial, such as providing non-mandatory professional development opportunities for staff rather than establishing a GSA that would directly involve students.

Many participants had knowledge or experiences of pushback from school leadership, parents, and community members to prior school-based initiatives to support LGBTQ+ students. Their recollections of such pushback revealed how community sentiments influenced the school leadership's use of discursive power. Such recollections also influenced how participants conceptualized their roles and power in school and community hierarchies while discouraging them from modifying aspects of school climates. For instance, an IRT lead at an urban school expressed worry about the professional consequences of involvement with efforts considered “controversial” in the community, citing backlash resulting in job loss for a teacher who tried starting a GSA 25 years prior. At another urban school, an IRT member cited ill-fated efforts some years back to advocate for a gender-neutral dress code for prom. Vocal community members protested, prompting increased enforcement of the old dress code by the school administration. This participant stated that the protest led to her professional marginalization in the school district: “I've paid a price. I'm stuck here. I won't ever move anywhere up.” In sum, it was common for knowledge and experience of past events to temper participants' perceptions of what types of changes were possible and thus their motivation to implement the EIPs.

Some LGBTQ+ participants anticipated negative responses to their efforts to implement EIPs. For example, a self-identified lesbian teacher at a small urban school expressed wariness that being involved would be perceived as personally motivated or self-serving. She stated:

A challenge for me is just always that…because I'm out, right, I'm seen as like, ‘Oh, this is a personal agenda,' and of course, it's deeply personal, but I'm not sure that it is an agenda, and I just know that it's like, these are the things that that have proven to work, right? So, this is what we're doing to get this result and to do what's right for kids.

This participant's tentativeness to become an implementation champion was grounded in her experience as a sexual minority and intimate knowledge of how heteronormative discursive power might negatively affect others' perceptions of her involvement.

Concerns about parental backlash influenced how schools approached EIP implementation. In some cases, the power exerted by communities was successful, as illustrated by an IRT in a conservative urban area that wanted to post sexual and reproductive health resource flyers in school bathrooms. Members approached the principal, who requested that they obtain approval from the parent-teacher association (PTA). After discussing the matter, most parents in the PTA were open to making the information available, conveying the perspective that “other” children—not theirs—might need it. Yet, one upset parent threatened to make a huge scene. As a result, the PTA denied the IRT's request, and the principal followed suit.

In other cases, participants sought to circumvent the influence of communities through their selection of EIP elements and the scale at which they implemented them. A principal at a suburban school described a need to help LGBTQ+ students without drawing the attention or ire of parents or the community. He expressed support for the EIPs and wellbeing of LBGTQ+ students but raised concern that the surrounding community was deeply religious and upheld conservative and traditional values. Moreover, the school district had no established policy regarding supportive practices for LGBTQ+ students. The principal stated,

Whatever gains we make, like the group [GSA] and these kinds of services we provide, we're trying to do them carefully. We're trying to make sure we don't expose our LGBTQ population to a potential problem or a negative reaction. And so we're very careful about ensuring that we're not exposing them to that kind of potential harm.

The sentiment of not wanting to place LGBTQ+ students in harm's way was common, especially in the study's first year, and was often used to rationalize hesitation or inaction related to EIPs.

Other participants expressed apprehension about opposition from parents and communities. However, they proceeded with EIP implementation, reasoning that the benefits for students outweighed the anticipated repercussions. The participants from a small rural school wanted to start a GSA but fretted over possible criticism from parents and the community. They ultimately decided to move forward and reportedly received no complaints despite their fears. In fact, the GSA drew students and was quite successful. When asked how families or community members responded to IRT actions at another school, the administrator said:

Well, for the most part, I think positive... And then I've also heard of a parent complaining and saying something to the administration along the lines of, ‘You might as well take that American flag down and put the Gay Pride flag up.'

Community pushback was not necessarily an insurmountable obstacle to change. For example, although an IRT at an urban school that worked to implement non-gendered security entry lines received complaints from some parents, the principal defended the policy and kept it in place. In another urban school, an IRT organized a suicide prevention presentation that included a video and a panel discussion on supporting LGBTQ+ students. Afterward, an IRT member described pushback from parents who thought the LGBTQ+ focus was “too much” but clarified that the principal was “extremely supportive and she's never afraid to stand up to a parent.” Rather than short-circuiting implementation, directly challenging the adverse reactions of parents and other community members could make it possible to move forward with new initiatives.

These findings illustrate how community influence on school contexts can affect implementation. For some school staff, experiences and expectations of failures and pushback informed their perceptions of the risk and potential for success in implementing LGBTQ+ supportive practices; and in some instances, standing up to community and parent disagreement could assist in carrying implementation forward. To varying degrees, community and families influenced what the IRTs could accomplish, as did leadership, as described below.

### Power of leadership

The significant power of administrators (e.g., principals, assistant principals) in school hierarchies influenced the motivation and ability of staff to take action to implement EIPs. Administrators were gatekeepers to material power (e.g., time, space, funding resources). Typically, those who were unsupportive cited community pressure, resource constraints, and individual beliefs and attitudes about the necessity and feasibility of implementing EIPs.

The barriers presented by unsupportive administrators necessitated adaptations by the IRTs, such as changing which administrator they partnered with or prioritizing smaller actions within their spheres of influence. In one rural school, the IRT and its coach determined that the principal who originally agreed to the study did not support the EIPs because of their explicit focus on LGBTQ+ students. Upon the coach's recommendation, the IRT enlisted the support of another school administrator identified as having more time and interest in their work. Going forward, the members reported greater communication and support from leadership and progress toward implementing EIPs, including establishing a GSA.

In an urban school with non-responsive leadership, participants consulted with their coach about ignored requests, emails, and other efforts. Even their implementation coach's outreach was met with non-response. Despite the discouraging disengagement, the IRT independently organized well-received after-school educational presentations for school staff, reaching out to community-based intermediary organizations to deliver the content.

In many instances, participants worked around obstacles created by leadership. However, formal power configurations in schools meant administrators had final decision-making authority. Unfortunately, this authority sometimes lent itself to schools not being able to move forward with implementation efforts either of specific practices or the entire project. The participation of one school in a rural conservative community prematurely ceased when a new superintendent unilaterally opposed school involvement in efforts to address the needs of LGBTQ+ students and instructed the principal to withdraw despite the interest and commitment of school staff. The nurse at this school expressed her disappointment, saying:

I'll even call them [students] and talk to them if I haven't seen them in a while. I will call them out [of class] and talk to them, but now the principal is telling me to leave it alone. The thing is that as a school nurse, he can't tell me what to do and what kids to see. I have a right to see any student, any student has a right to see me, they have a right to talk about anything they want to, and the principal has no say.

In contrast, many school administrators leveraged discursive and material power to support IRTs, enabling them to make changes beyond their scopes of influence, such as adjusting school policy, initiating structural changes to the environment, or fielding opposition from the staff and community. The assistant principal overseeing facilities in an urban school was instrumental in helping the IRT establish a single-user, gender-neutral restroom and brainstorming strategies to make behavioral, sexual, and reproductive health information readily available to students at on-campus resource hubs.

In addition to directly supporting change within schools, administrators defended the EIPs against critics. On the topic of professional development related to transgender students, IRT members at an urban school offered examples of the principal's support:

[The principal] had no problem making sure that all of our staff participated in that training…. We did have a couple of hecklers in there. She was willing to make sure that that didn't distract from what we needed to know in order to keep all of our students safe and providing that information to all of our teachers.

In another urban school, students supported by the IRT endeavored to change the school's homecoming court, which typically featured a “king” and “queen,” by designating the winners of the competition as “royalty.” This change, however, spurred some parents to complain. Aware of the nature of the student-led change, the principal assumed the responsibility of addressing parental demands to retain the traditional court. IRT members agreed that the principal took a rather radical stance in the context of this community by defending the new terminology and that students and staff were grateful to know that she “had their back.”

School leadership's support was not static, with many growing into their willingness and ability to foster implementation over time. In some cases, education on the needs of LGBTQ+ youth in their schools catalyzed major shifts in administrative support. The principal of a school in a conservative rural community espoused the belief that their school was safe for LGBTQ+ students. This school's IRT supported a gender-diverse student in sharing with the principal their experience of fearing violence in school and “being jumped on the way home.” Both the principal and IRT described this conversation as a “game-changer” for implementation. Seeing the school through a student's eyes made the principal shift gears to support EIPs to nurture the safe and supportive environment she had presumed existed. We observed such shifts in other schools over time, albeit ones that tended to be more subtle in their manifestation. Administrators in the early years of RLAS generally lacked awareness of LGBTQ+ student needs, including their higher risk for suicidal behaviors. This starting point contrasts with interviews in later years when the same administrators displayed a strong understanding of the differential and increased support needs of LGBTQ+ students. This instance illustrates how epistemic power generated through the IRT highlighted otherwise unknown perspectives of students to shift the discursive power of leadership in their favor.

Similar to staff turnover, instability in school leadership could compromise implementation progress. New administrators were often overwhelmed by their positions and had insufficient familiarity with schools, which rendered them unhelpful to the IRTs. Yet, turnover in leadership also introduced IRTs to new allies. In one urban school, 2 years of repeated requests to transition single-stall staff restrooms to gender-neutral restrooms accessible to students finally received a positive response when an incoming principal quickly agreed to the change. An IRT member praised his receptiveness, referring to it as “incredible, incredible” compared to “the first couple years of the grant that we were running into walls when it came to the gender-neutral bathrooms.” The IRT member added, “That was a two-year battle, and then this guy came in, and poof, it just happened.” Similarly, the new principal agreed to make time for all-staff professional development that had also been stalled for 2 years, enabling the IRT to organize training on LGBTQ 101 and Safe Zones for 50 school personnel. This change in administration further demonstrated the consolidation of discursive and material power at the top tiers of school leadership and the role of administrators in constraining or enabling action.

Many participants suggested that aligning themselves with RLAS itself was empowering. They felt that their status as IRT members gave them some power as the study was perceived to have official expectations beholding the school. One IRT member stated, “Now that we're overseeing this program, I feel more of a responsibility to make sure that I know what's going on with their GSA club and everything else going on throughout our whole campus.” This sentiment was echoed by multiple other participants, underscoring the potential and importance of generating discursive and epistemic power within involved staff members.

While formal leadership could easily constrain IRT actions through lack of buy-in and the use of their considerable discursive and material power, the IRTs able to negotiate and leverage the power of administrators in their schools found the most success with implementation. Leadership could garner the necessary resources for innovations and use their authority to challenge negative pushback from community and staff members.

### Power of policy

Participants varied in their perspectives on the power of policy and their power over policy. We documented several key dynamics regarding policy in participating schools. First, some IRTs deprioritized policy implementation, including one of the six EIPs on bullying and harassment prohibitions, because they believed their schools' current practices were safe and supportive. Second, policies already explicitly including protections for sexual orientation and gender identity and expression led participants to believe their schools had fully implemented this EIP, regardless of the absence of follow-through, training, or other mechanisms to translate policy into practice. Similarly, new policies at the state level had the potential to impact all schools, but a lack of dissemination and enforcement stifled change. Participants sometimes hesitated to address policy, asserting it did not fall within the purview of their roles vis-à-vis the school or the district. In contrast, several IRTs initiated reviews of current policies at the school and occasionally district levels and then took action to change them. These dynamics provide insight into participants' perceptions about their ability to initiate policy change at an institutional level and the discursive power of policy to perpetuate or disrupt structural stigma.

Participants' lack of awareness of details regarding bullying and harassment policies, restroom rules, dress codes, and gender support plans (official accommodation agreements for transgender and gender-diverse students), often shaped their perceptions of how protective school policies were of LGBTQ+ students. For example, while participants might believe that a bullying policy protecting all students existed, they were unaware of its specifics or possible deficits. An IRT lead stated, “As far as our policy, I'm not sure, but I'm assuming that it's in there that we treat everybody equally.” Administrators espoused similar views, often claiming that existing bullying policies protected all students, including LGBTQ+ youth.

Administrators and other participants reasoned that existing bullying and harassment prohibitions that did not enumerate sexual orientation or gender identity and expression were sufficient to support LGBTQ+ students. They cited as evidence the fact that students and faculty reported bullying to the administration. For example, one administrator reasoned, “Bullying is bullying, right? And all different types of students experience bullying, so we do have our bullying policy. I think our students feel safe reporting bullying incidents.” Other participants cited their current practices as evidence against adopting more robust policies. A second administrator explained, “We don't have specific policies, written policies. They're just unwritten rules that we have in the school that people are aware of.” Similarly, a third administrator said, “We've always worked case by case, individual by individual, and work to find the best solutions with that. And so, yeah, I've worked in the absence of policy, but I also felt that we worked—and I've worked, certainly—with the best interest of the students.”

Without an official policy, attempts to implement changes in schools sometimes boiled down to the whims of school leadership, which was frustrating for participants. In one rural conservative community, the school permitted students to change their names on identification cards, and the administration encouraged teachers to use students' chosen names and gender pronouns. There was no written policy—it did not seem necessary to staff since the school climate was welcoming and supportive. However, when school leadership changed significantly, the new principal single-handedly ended the practice and even advised teachers against using chosen names and pronouns in their student interactions. IRT members claimed they had nothing to stand on to influence the administration to change its stance as no official policy was in place regarding chosen names and pronouns.

Participants at some schools recognized the importance of policy change to enable supportive action and safeguard implementation initiatives over time. One IRT lead sharing this perspective said, “If we don't put policy in, then when those of us who are here and doing trainings and working with staff leave, then you go back to the old ways. So, policy has to happen to keep things moving forward.”

The extent to which communication regarding new or existing policies occurred in school communities was questionable, given delays in sharing information and insufficient awareness. An IRT member involved in school policy change underscored the importance of communicating the new policies to staff and students and expressed concern that students may lack awareness:

The word isn't getting out. What I'm going to propose now is that once staff gets that weekly bulletin with the school policies, that they start reading them to kids in the morning and then talking about it, ‘What do you think this means?' That's my next step.

One IRT successfully changed bullying and harassment policies at its school to explicitly protect LGBTQ+ students. However, over the study's course, this IRT's school experienced significant staff turnover that hampered effective communication about their content. Consequently, newer staff, including IRT members and students, were commonly unaware of the revised policies. Without communication, the revised policies fell short in supporting LGBTQ+ students.

In 2019, the New Mexico legislature passed the pivotal “Safe Schools for All Students” Act, requiring public schools to adopt bullying policies with explicit protections for LGBTQ+ students (69). However, we found that many study participants were unaware or only marginally aware of the legislation in the two school years following its enactment. Some IRT members with knowledge of the legislation attempted to offer trainings and disseminate information on the new policy. They had varying degrees of success, depending on how proactive their school districts were in supporting and sharing information about the legislation.

In conversation with implementation coaches, participants tended to rate the feasibility of policy change related to bullying and harassment lower than other EIPs, such as establishing safe spaces or facilitating professional development for school staff. An IRT member explained that during their first year of implementation, the team had relative ease organizing professional development and safe spaces. However, when this member looked ahead to the next school year, they described impacting policy as daunting: “Now I'm like, ‘Oh my god, policy?' Like how are we—? That's intimidating.” Participants sometimes characterized policy formulation as the responsibility of high-ranking school leadership or the school district rather than a process they could readily initiate.

Despite challenges associated with establishing and enacting protective policies, which typically resulted in the deprioritization of policy implementation, IRTs still found considerable success. For example, bolstered by IRT assistance, a student-led GSA fruitfully advocated for a district-wide policy supporting the use of chosen names in virtual classrooms during the COVID-19 pandemic. They also lobbied the school board to formally affirm LGBTQ+ student rights in response to proposed bans on transgender athletes in high school sports. An administrator noted that in taking these actions, “It puts the school board on record of saying we support all of these initiatives to really accept unified support [and] provide resources for our LGBTQ community.”

## Discussion

Our findings demonstrate the importance of understanding how power operates in and across outer and inner contexts to bound, shift, amplify, and otherwise shape the way new practices are received, implemented, and sustained. Heteronormativity and the structural stigma engendered by it are forms of dominant power exerted through institutions like schools, perpetuating adverse health outcomes for LGBTQ+ youth and constraining intervening actions. Stigma scholars point to structural stigma as the fundamental cause of population health inequities (17, 70, 71). On the individual level, structural stigma contributes to the psychological processes of minority stress through such mechanisms as experiences of discrimination or concerns about concealment and disclosure of identity (15, 22, 31, 72). Institutional spaces like schools, as part of their function for (re)producing heteronormative subjects, generates and sustains structural stigma that then impacts the health and wellbeing of LGBTQ+ young people.

Heteronormativity represented a pervasive form of discursive power that shaped the perceptions, expectations, and practices of students and school staff—including their aspirations for change—as well as the norms, rules, and institutional structures in which they operated. In this way, heteronormativity and structural stigma influenced how epistemic, discursive, and material power functioned in schools, thus leading us to conceptualize the work of IRTs as an exercise in resistant power.

In their work addressing LGBTQ+ equality in primary schools in the United Kingdom, education scholars Renee DePalma and Elizabeth Atkinson distinguish between anti-homophobia work (i.e., rules against bullying and policies prohibiting discrimination based on sexual orientation or gender identity) and counter-heteronormative work (i.e., resisting and restructuring dominant standards for appropriate or normal forms of sexuality and gender) (13). Our study resonates with DePalma and Atkinson's critique that policy alone cannot instantiate deep, sustainable change. However, our study illustrates that policy is critical to conferring the power and confidence to engage in counter-heteronormative efforts. Strengthening policy can be a sustainable way to institutionalize LGBTQ+ affirmation, inclusivity, and protections, outlasting the involvement of any individual or group of individuals; thus, supporting on-the-ground actors in challenging critics who wish to prevent or eliminate LGBTQ+ supportive practices.

Our findings have several implications for implementation science research and practice. Implementation science has traditionally noted the influence exerted by macro forces (e.g., legislation, funding) on inner contexts and the need to adapt implementation strategies in response (51). However, RLAS elucidates that power is also diffuse, fluid, and discursive. The pervasiveness of heteronormativity and structural stigma poses a challenge to implementation scientists by forcing us to think beyond the bounds of simple and discrete constructs. In many extant frameworks, heteronormativity would likely be categorized under constructs like “sociopolitical context” or “culture.” In the EPIS framework, “sociopolitical context” is chiefly a part of the outer context and “culture” is relegated to the inner context (55, 56). The Consolidated Framework for Implementation Research (CFIR) traditionally places culture (commonly thought of as subconstructs of stress and effort) at the organizational level of the inner context. It accounts for individuals' relationships to and attitudes about innovations but not necessarily their attitudes about the populations for which the intervention is meant to benefit (73–75). The integrated-Promoting Action on Research Implementation in Health Services (i-PARIHS) framework recognizes the interconnected multilayered nature of inner and outer contexts. Yet it also conceptualizes culture as primarily within the inner context of organizational implementation sites (76). None of the models accurately reflect how heteronormative thinking can shape the discursive, epistemic, and material power involved in the RLAS implementation at every level and stage. For example, the acceptability, appropriateness, and feasibility of EIPs often hinged on how heteronormativity shaped participants' perceptions and, therefore, how they used epistemic and discursive power toward or against implementation. For many, it was not outright homophobia or transphobia that made it challenging to implement LGBTQ+ supportive practices, but the belief that schools were already doing enough to support all students or an assumption that specialized supportive intervention would be perceived as “special treatment (12).” Consequently, common implementation science constructs, such as appropriateness, acceptability, and feasibility, encompass more than individual attitudes; they also reflect the institution and wider social context in which interventions are to be implemented.

The powerful influence of parents as bridging factors linking inner and outer contexts, accentuates the need to consider how such factors can positively and negatively influence implementation processes (58, 77–79). Communities exert heteronormative disciplinary power. As described by Foucault, disciplinary power is the chief mechanism through which modern power systems bring subjects into line with dominant standards—in this case, heteronormative standards (80). In some instances, this power is exercised through overt discrimination. It is expressed as resistance to change in others. As bridging factors between communities and schools, parents leverage this power over their children and schools through disagreement with LGBTQ+ supportive innovations. Alternatively, they potentially positively influence implementation efforts by supporting interventions.

Implementation science work on health equity offers a corrective to such overly simplistic conceptualizations by encouraging greater attention to how higher-level social determinants impact clinical encounters (81, 82). For example, the Health Equity Implementation Framework conceptualizes “societal influence” as shaping context, recipient, and innovation factors and explicitly calls out insidious influences such as racial bias (81). Taking the recognition of structural causes further, the recent race(ism)-conscious adaptation of the CFIR problematizes “race-neutral” construct definitions to show the cross-construct operation of racism and racialization impacting implementation (83). Heteronormativity, even if it does not appear as overt homophobia and transphobia, operates similarly to how this model describes racism. Scholars in the field have explicitly called for implementation science to identify the root causes of health disparities, such as structural racism and other oppressive power dynamics, as critical to addressing barriers to implementation. These approaches stress the need for formative research to understand the influence of structural causes, the involvement of invested stakeholders, and multilevel and multicomponent strategies to mitigate the impacts of these root causes (82, 84). The findings of this study vividly illustrate the importance of these efforts.

As Foucault asserted, power's diffuse nature means that it is not held exclusively by any single person or group; its relational nature implies that both resistance and dominance operate within the same space (3). The role that RLAS played in cultivating resistant power in the top-down hierarchies of school environments further highlights a need to complicate understandings of power in implementation science contexts is. Our findings show that participants were often able to negotiate and incorporate extant power hierarchies in schools to work toward implementation goals. Many IRTs successfully recruited administrators and other power brokers in their schools to support their efforts, even when these efforts resisted heteronormativity and ensconced school operations. These findings underscore the usefulness of garnering leadership buy-in and active support, which implementation science often highlights (85–88), and further emphasize the significance of leadership alignment when interventions are focused on marginalized populations (89, 90).

Our results also highlight the crucial role of education as a form of resistant discursive and epistemic power leading to the leveraging of material power (51, 91). First, education on LGBTQ+ topics (e.g., disparities) helps frame the necessity of EIPs, countering dominant narratives informed by heteronormativity that all students should be treated the same and establishing a knowledge base from which implementers can act. Professional development about the challenges LGBTQ+ youth face can garner sympathetic support from school staff and improve buy-in for implementation (91, 92). Second, education can help highlight “subjugated knowledges” or other voices in school contexts that would normally not be valued. Subjugated knowledge or situated knowledge is highly contextual and local. This knowledge can be contrasted with the standardized knowledge circulating within disciplinary spaces like schools (93). For example, youth voices in our study were able to shift administrators' views about the safety and supportiveness of their schools.

Implementation science has recognized the importance of education in the implementation process (94–96). Still, researchers should pay closer attention to how education can highlight situated knowledge to create resistant power (2). What participants know about their lives, experiences, and social worlds can directly contradict knowledge generated elsewhere, offering innovative solutions to difficult problems, exposing interventions or strategies that will not work in practice, or countering narratives that frame health problems in ways that do not align with reality. Participants were able to counter administrators who claimed that the school already supported “all students” equally. Strategies that validate the knowledge of people possessing intimate understanding of schools and people inhabiting LGBTQ+ identities can reverse the dynamics of dominant discursive and epistemic power that contribute to the erasure of their identities and experiences, obviating the need for action in the first place. Strategies that use this resistant discursive and epistemic power can also improve the chances of successful implementation through effective adaptation and tailoring.

Relatedly, while implementation science has recognized the significance of champions in garnering buy-in for new interventions (75, 97, 98), our findings show that cultivating champions in implementation studies can offer participants a sense of empowerment and legitimization that helps them resist the constraints traditionally placed on their roles. Participants' sentiments expressing the empowering nature of being a part of this study suggest that the experience of leadership changed participants' perceptions of their power in schools. Some participants expanded their purview by proactively becoming knowledgeable and aware of what was going on beyond their immediate roles on campus. In so doing, they also gained confidence and a sense of satisfaction that they could make changes that mattered to students. By generating resistant discursive and epistemic power within implementation contexts through education, leadership alignment, and champions, IRTs were able to access the material power necessary for implementation.

## Conclusion

Implementation scientists must consider the real and perceived power differentials affecting an organization's readiness to implement an intervention or an individual's motivation to invest in implementation. Concepts such as readiness and motivation implicate individual, relational, structural, and broader contextual factors. Improving the likelihood of successful implementation requires recognizing these factors and addressing the full ecology of the implementation environment to avoid an over-emphasis on individual capabilities and efforts and dismissing implementation challenges as the consequence of individual or team failings.

Finally, in promoting efforts to improve health equity, implementation scientists must support implementers in leveraging resistant power to counter the institutional structures and social norms that perpetuate inequities, like heteronormativity. Implementation scientists and practitioners need to think beyond the fit of interventions with contexts to consider the productive nature of interventions that challenge and disrupt—or otherwise do not fit institutional processes. The challenges facing such efforts are formidable in environments shaped by hierarchical governance structures that control material power, such as schools. In these contexts, deploying strategies that generate and leverage resistant discursive and epistemic power may be key to obtaining material power for resistant purposes. Strategies like cultivating champions, education and training, building capacity, aligning with leadership, and enacting policy are practical ways to bolster resistant power in schools to support and sustain EIPs.

## Data availability statement

The data that support the findings of this study are available from the study Principal Investigator (CW) upon reasonable request.

## Ethics statement

The studies involving human participants were reviewed and approved by the Pacific Institute for Research and Evaluation Institutional Review Board. The patients/participants provided their written informed consent to participate in this study.

## Author contributions

DS and CW conceived the present analysis. CW is the co-principal investigator for the parent RLAS study from which the data were drawn. DS, BR, EJ, and CW conducted primary data collection. EB helped organize fieldwork and conducted literature reviews. BR conducted qualitative analysis and constructed school reports. DS, BR, and EJ led the writing of the manuscript. All authors contributed to the analysis, interpretation of findings, and reviewed and edited multiple versions of the manuscript. All authors contributed to the article and approved the submitted version.

## Funding

This work was funded by the Eunice Kennedy Shriver National Institute of Child Health and Human Development (1R01HD83399).

## Conflict of interest

The authors declare that the research was conducted in the absence of any commercial or financial relationships that could be construed as a potential conflict of interest.

## Publisher's note

All claims expressed in this article are solely those of the authors and do not necessarily represent those of their affiliated organizations, or those of the publisher, the editors and the reviewers. Any product that may be evaluated in this article, or claim that may be made by its manufacturer, is not guaranteed or endorsed by the publisher.
